# Drug-Eluting Stents in Patients with Chronic Kidney Disease: A Prospective Registry Study

**DOI:** 10.1371/journal.pone.0015070

**Published:** 2010-11-29

**Authors:** Chetan Shenoy, Judy Boura, Pamela Orshaw, Kishore J. Harjai

**Affiliations:** 1 Guthrie Clinic, Sayre, Pennsylvania, United States of America; 2 William Beaumont Hospital, Royal Oak, Michigan, United States of America; University of Turin, Italy

## Abstract

**Background:**

Chronic kidney disease (CKD) is strongly associated with adverse outcomes after percutaneous coronary intervention (PCI). There are limited data on the effectiveness of drug-eluting stents (DES) in patients with CKD.

**Methodology/Principal Findings:**

Of 3,752 consecutive patients enrolled in the Guthrie PCI Registry between 2001 and 2006, 436 patients with CKD - defined as a creatinine clearance <60 mL/min - were included in this study. Patients who received DES were compared to those who received bare metal stents (BMS). Patients were followed for a mean duration of 3 years after the index PCI to determine the prognostic impact of stent type. Study end-points were all-cause death, myocardial infarction (MI), target vessel revascularization (TVR), stent thrombosis (ST) and the composite of major adverse cardiovascular events (MACE), defined as death, MI or TVR. Patients receiving DES in our study, by virtue of physician selection, had more stable coronary artery disease and had lower baseline risk of thrombotic or restenotic events. Kaplan-Meier estimates of proportions of patients reaching the end-points were significantly lower for DES vs. BMS for all-cause death (*p* = 0.0008), TVR (*p* = 0.029) and MACE (*p* = 0.0015), but not MI (*p* = 0.945) or ST (*p* = 0.88). Multivariable analysis with propensity adjustment demonstrated that DES implantation was an independent predictor of lower rates of all-cause death (hazard ratio [HR] 0.48, 95% confidence interval [CI] 0.25–0.92), TVR (HR 0.50, 95% CI 0.27–0.94) and MACE (HR 0.62, 95% CI 0.41–0.94).

**Conclusions:**

In a contemporary PCI registry, selective use of DES in patients with CKD was safe and effective in the long term, with lower risk of all-cause death, TVR and MACE and similar risk of MI and ST as compared with BMS. The mortality benefit may be a result of selection bias and residual confounding, or represent a true finding; a hypothesis that warrants clarification by randomized clinical trials.

## Introduction

In patients with coronary artery disease, the presence of chronic kidney disease (CKD) is strongly associated with increased mortality and an increased incidence of major adverse cardiovascular events (MACE) after percutaneous coronary intervention (PCI) [1], [2], [3].

Coronary stents have substantially improved the efficacy of percutaneous revascularization. Drug-eluting stents (DES) have further reduced the rates of in-stent restenosis and repeat revascularization compared to bare metal stents (BMS) [4], [5], [6]. However, there are limited data on the safety and efficacy of DES in patients with CKD because these patients are systematically excluded from major interventional cardiology trials [7], [8], [9].

The purpose of the current study was to determine the long-term effectiveness of DES in patients with CKD. Using a prospective registry of consecutive patients undergoing PCI at our tertiary care academic hospital, we evaluated clinical outcomes following DES use compared with BMS use in patients with CKD.

## Methods

### Ethics

Data were analyzed anonymously and informed consent was neither required nor obtained. Guthrie Clinic's Institutional Review Board approved the study and agreed that informed consent was not required. Approval was neither required nor obtained from ethics committees.

### Patient Population

The Guthrie PCI Registry is a prospective, observational registry of all patients undergoing PCI at our center. As described previously [10], [11], demographic, clinical, angiographic, laboratory values and in-hospital outcomes are collected by dedicated specially trained nursing personnel, in a standardized fashion in accordance with American College of Cardiology National Cardiovascular Data Registry (ACC-NCDR) definitions [12]. Longitudinal follow-up information is obtained annually using multiple concurrent approaches including patient follow-up visits, surveillance of medical records, contact with primary care providers, telephone contacts with registry participants or next-of-kin, and linkage with the Social Security Administration Death Master File.

**Figure 1:**
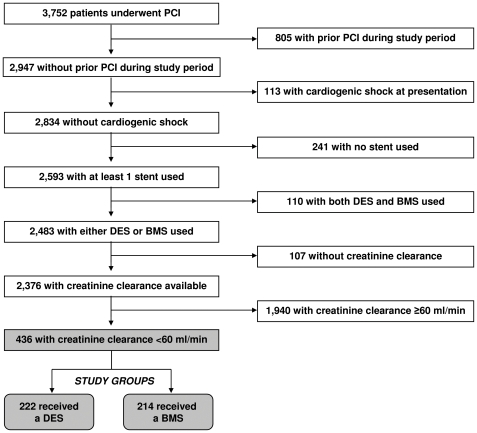
Study Patients. BMS  =  bare metal stent; Creatinine clearance  =  creatinine clearance; DES  =  drug-eluting stents; PCI  =  percutaneous coronary intervention.

3,752 patients underwent PCI procedures at our center and were enrolled in the Registry between January 2001 and December 2006. Patients were excluded from the current study if they had undergone any prior PCI at our institution during the study period, were diagnosed with cardiogenic shock prior to PCI, had not received a stent or had received a combination of DES and BMS. Patients were also excluded if their baseline creatinine was not available. Based on these exclusion criteria, 2,376 patients remained, of which, 436 had a creatinine clearance of <60 mL/min and constituted the cohort for the current study (Figure 1). Study subjects were assigned to either the DES group or the BMS group according to the stent type used during the index PCI.

### Assessment of Renal Function

Baseline creatinine values obtained before the index PCI were used to calculate creatinine clearance according to the Cockcroft-Gault formula: creatinine clearance (milliliters/minute)  =  [(140-age) X weight (kilograms)]/[serum creatinine (milligrams/deciliter) x 72], corrected in women by a factor of 0.85 [13]. A creatinine clearance of <60 mL/min, corresponding to at least moderate CKD as per the National Kidney Foundation guidelines [14], was used to define patients with CKD for the purposes of this study. While the Modification of Diet in Renal Disease (MDRD) formula is more accurate than the Cockcroft-Gault formula in estimating glomerular filtration rates <60 mL/min, the Cockcroft-Gault formula was used for assessment of renal function since it has been shown to identify more patients at risk for adverse clinical outcomes following non-ST-segment elevation acute coronary syndromes compared to the MDRD formula [15].

### Prevention of Contrast-mediated Renal Dysfunction

At our institution, patients deemed at risk for contrast-mediated renal dysfunction are treated with intravenous sodium bicarbonate solution (150 mEq sodium bicarbonate in 1000 ml 5% dextrose in water) at 3 ml/kg/hour for 1 hour prior to the PCI, and at 1 ml/kg/hour for 6 hours following the PCI. N-acetylcysteine 600 mg twice a day for 4 doses is also used in combination with intravenous sodium bicarbonate solution. Our protocols recommend prophylactic treatment for all patients with estimated glomerular filtration rates <60 mL/min. Patients undergoing urgent or emergent PCI or those who are deemed unsuitable for aggressive hydration (due to left ventricular dysfunction, congestive heart failure or uncontrolled hypertension) receive abbreviated regimens of intravenous sodium bicarbonate and N-acetylcysteine.

### Definitions

As outlined in the ACC-NCDR [12], procedures were classified as elective or non-elective based on the presentation (stable angina vs. unstable angina, NSTEMI or STEMI) and risk of infarction or death. Elective procedures could have been performed on an outpatient basis or during a subsequent hospitalization without significant risk of infarction or death. For stable inpatients, elective procedures were performed during the hospitalization for convenience and ease of scheduling and not because the patients' clinical situation demanded the procedure prior to discharge. All other procedures were classified non-elective. The study outcomes were all-cause death, myocardial infarction (MI), target vessel revascularization (TVR), stent thrombosis (ST) and the composite of major adverse cardiovascular events (MACE), defined as death, MI or TVR. Standard definitions, outlined in the ACC-NCDR [12], were used to define peri-procedural complications. MI was defined as the occurrence of 2 or more of the following: chest pain, abnormal electrocardiographic changes suggestive of acute myocardial infarction, or elevated cardiac biomarkers. TVR was defined as PCI performed in a vessel treated during the index procedure or any coronary artery bypass grafting procedure performed after the index procedure, due to recurrence of angina or other evidence of ischemia. ST for the purposes of the current study included either definite or probable ST as defined by the Academic Research Consortium [16].

### Statistical Analysis

Continuous data are presented as mean ± standard deviation. Wilcoxon rank tests were used to compare mean values of continuous data between 2 groups. Categorical variables are reported as percentages and comparisons were made using Chi-square tests where appropriate (expected frequency >5), otherwise Fisher's Exact tests were used. Using the Kaplan-Meier method, event-free survival curves were constructed for all study outcomes and compared using log-rank tests. Because the patients were not randomly assigned to receive either DES or BMS, the propensity to receive a DES was estimated by performing multivariate logistic regression using all baseline variables that showed a univariate association (*p*<0.10) with DES implantation. To assess the impact of choice of stent type on all-cause death, TVR and MACE, we performed Cox proportional hazards regression analyses. Duration of follow-up was truncated at 4 years (1,460 days). In multivariable Cox regression analyses, the initial model included stent type (DES vs. BMS), propensity score for receiving DES and all variables that showed a significant relation with stent type (*p*<0.10). Backward stepwise regression was performed, eliminating variables that showed no significant association (*p*>0.05) with the outcome. The variable denoting stent type was retained in the model irrespective of its statistical significance. In the final models, we estimated adjusted hazards ratios (HR) and 95% confidence intervals (CI) denoting the impact of stent type on the study outcomes. All statistical tests were 2-tailed and *p*<0.05 was regarded as significant. All analyses were conducted using The SAS® System for Windows version 9.1.3, Service Pack 4, Cary, North Carolina, USA.

## Results

Of 2,376 consecutive all-comer patients enrolled in the Guthrie PCI Registry with a baseline serum creatinine measurement, index procedure during the study period, without cardiogenic shock and implantation of either DES or BMS, 18% (436) had a creatinine clearance of <60 mL/min. Of these 436 patients, 222 (51%) patients had received DES and 214 (49%) patients had received BMS.

### Clinical, Procedural, and Angiographic Characteristics

Tables 1 and 2 present the clinical, and procedural and angiographic characteristics, respectively, of the study patients, stratified by the study group.

**Table 1:** Clinical Characteristics.

Characteristic	All(*n* = 436)	BMS(*n* = 214)	DES(*n* = 222)	*p*-Value
Age, years	71+/−10	73+/−10	69+/−11	**0.0003**
Male	87 (20%)	49 (23%)	38 (17%)	0.13
Weight, kilograms	69+/−11	67+/−11	70+/−12	**0.0044**
Body surface area, meters squared	1.73+/−0.15	1.71+/−0.16	1.75+/−0.15	**0.015**
Diabetes mellitus	90 (21%)	33 (15%)	57 (26%)	**0.0082**
Cigarette smoking	100 (22%)	48 (22%)	52 (23%)	0.61
Hypertension	296 (68%)	143 (67%)	153 (69%)	0.64
Dyslipidemia	297 (68%)	133 (62%)	164 (74%)	**0.0086**
Peripheral vascular disease	58 (13%)	30 (14%)	28 (13%)	0.67
Cerebrovascular disease	37 (8%)	21 (10%)	16 (7%)	0.33
Prior MI	81 (19%)	47 (22%)	34 (15%)	0.074
Prior CABG	65 (15%)	37 (17%)	28 (13%)	0.17
Prior PCI	57 (13%)	25 (12%)	32 (14%)	0.40
Heart failure	35 (8%)	19 (9%)	16 (7%)	0.52
Non-elective procedure	270 (62%)	153 (71%)	117 (53%)	**<0.0001**
Presented with STEMI	79 (18%)	50 (23%)	29 (13%)	**0.005**
Presented with NSTEMI	111 (25%)	59 (28%)	52 (23%)	0.32
Received thrombolytic drug before PCI	25 (6%)	20 (9%)	5 (2%)	**0.0016**
Creatinine clearance, mL/min	47+/−9	46+/−10	47+/−8	0.17

*p*<0.05 for pairwise comparison between DES and BMS groups.

Continuous variables are expressed as mean ± standard deviation (median). Categorical variables are expressed counts (percentages).

BMS  =  bare metal stent; CABG  =  coronary artery bypass graft surgery; DES  =  drug-eluting stents; MI  =  myocardial infarction; NSTEMI  =  non-ST-segment elevation myocardial infarction; PCI  =  percutaneous coronary intervention; STEMI  =  ST-segment elevation myocardial infarction.

**Table 2:** Procedural and Angiographic Characteristics.

Characteristic	All(*n* = 436)	BMS(*n* = 214)	DES(*n* = 222)	*p*-Value
LVEF	49+/−12	47+/−13	50+/−11	**0.015**
Vessel diameter, mm	3.1+/−0.5	3.2+/−0.6	3.0+/−0.4	**0.0003**
Multivessel PCI	47 (11%)	27 (13%)	20 (9%)	0.22
Lesion length >28 mm	55 (13%)	34 (16%)	21 (9.5%)	**0.041**
Chronic total occlusion	6 (1.3%)	2 (0.9%)	4 (1.8%)	0.69
Bifurcation lesion	10 (2.2%)	2 (0.9%)	8 (3.6%)	0.11
Ostial lesion	23 (5.3%)	9 (4%)	14 (6%)	0.33
Restenotic lesion	8 (1.8%)	2 (0.9%)	6 (2.7%)	0.29
Use of IABP during PCI	19 (4.3%)	12 (6%)	7 (3%)	0.19
Use of GPI during PCI	335 (77%)	186 (87%)	149 (67%)	**<0.0001**
Successful PCI	414 (95%)	195 (92%)	219 (99%)	**0.0002**

*p*<0.05 for pairwise comparison between DES and BMS groups.

Continuous variables are expressed as mean ± standard deviation (median). Categorical variables are expressed counts (percentages).

BMS  =  bare metal stent; DES  =  drug-eluting stents; GPI  =  glycoprotein IIb/IIIa inhibitor; IABP  =  intra-aortic balloon pump; LVEF  =  left ventricular ejection fraction; PCI  =  percutaneous coronary intervention.

Patients who received DES were more likely to be have been younger (69 vs. 73 years; *p* = 0.0003), heavier (70 vs. 67 kg; *p* = 0.0044), diabetic (26% vs. 15%; *p* = 0.0082) and dyslipidemic (74% vs. 62%; *p* = 0.0086) than those who received BMS. They were also more likely to have had smaller vessels (3.0 mm vs. 3.2 mm; *p* = 0.0003), higher LVEF (50% vs. 47%; *p* = 0.015) and successful PCI (99% vs. 92%; *p* = 0.0002).

Patients who received DES were less likely to have presented with acute STEMI (13% vs. 23%; *p* = 0.005), had a non-elective procedure (53% vs. 71%; *p*<0.0001) and received thrombolytic therapy prior to the PCI (2% vs. 9%; *p* = 0.0016) than those who received BMS. They were also less likely to have had lesions longer than 28 mm (9.5% vs. 16%; *p* = 0.041) and received glycoprotein IIb/IIIa inhibitor drugs during PCI (67% vs. 87%; *p*<0.0001).

Mean creatinine clearance was not significantly different between the DES and BMS groups (47 vs. 46 mL/min; *p* = 0.17).

### Propensity score

Based on the univariate analyses, a step-down logistic regression analysis was done to create a propensity score for receiving DES instead of BMS. Included in the first step were all the variables from univariate analyses with *p*<0.10. The least significant variable was dropped at each step until only those variables with *p*<0.05 remained in the final model. Using the parameter estimates for creating a propensity score, the following formula was reached for the propensity score:

Propensity score  = 4.8383+ dyslipidemia x (0.5314)+ thrombolytic use x (−1.0176)+ use of glycoprotein IIb/IIIa inhibitor drugs x (−1.3345)+ age x (−0.0306)+ weight x (0.0246)+ vessel diameter x (−1.1752).

The *c* statistic was 0.73 and the Global WALD statistic was 58 with 6 degrees of freedom (*p*<0.0001).

### Outcomes

The mean follow-up period was 2.97±1.14 (median 3.39) years (range 0 to 4 years). No patients were lost to follow-up.

### Kaplan–Meier Analyses

**Figure 2:**
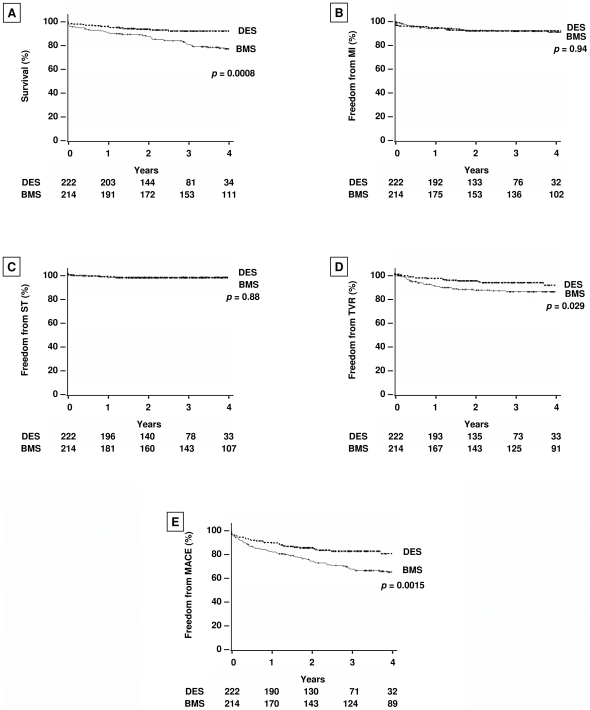
Kaplan-Meier Curves of Estimated Cumulative Incidence of Outcomes. The estimated cumulative incidences of all-cause death (A), MI (B), ST (C), TVR (D) and MACE (E) are shown. BMS  =  bare metal stent; DES  =  drug-eluting stents; MACE  =  major adverse cardiovascular events; PCI  =  percutaneous coronary intervention; MI  =  myocardial infarction; ST  =  stent thrombosis; TVR  =  target vessel revascularization.

Kaplan–Meier curves are shown in Figure 2. Compared to BMS patients, DES patients had significantly lower rates of all-cause death (*p* = 0.0008, Figure 2A) without lower rates of MI (*p* = 0.94, Figure 2B) or ST (*p* = 0.88, Figure 2C). DES patients also had significantly lower rates of TVR (*p* = 0.029, Figure 2D) and MACE (*p* = 0.0015, Figure 2E) compared to BMS patients.

### Multivariable Analysis

**Figure 3:**
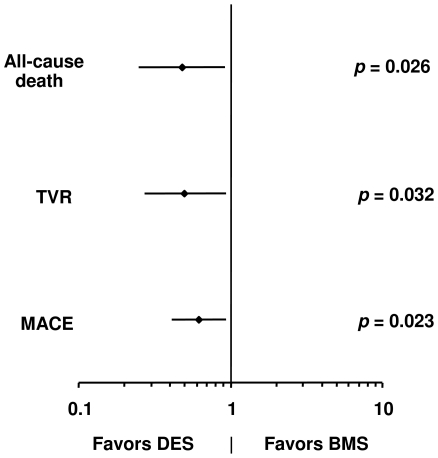
Multivariable Analysis with Propensity Adjustment Denoting the Impact of Stent Type on All-Cause Death, TVR and MACE. BMS  =  bare metal stent; DES  =  drug-eluting stents; MACE  =  major adverse cardiovascular events; TVR  =  target vessel revascularization.

Multivariable analysis with propensity adjustment (Figure 3) demonstrated that DES implantation was an independent predictor of lower all-cause death (hazard ratio [HR] 0.48, 95% confidence interval [CI] 0.25–0.92), TVR (HR 0.50, 95% CI 0.27–0.94) and MACE (HR 0.62, 95% CI 0.41–0.94).

## Discussion

We found that the use of DES in consecutive patients with CKD undergoing PCI was associated with improved outcomes in terms of all-cause death, TVR and MACE. We also found that DES were not associated with a higher risk of ST compared to BMS in these patients. Finally, DES were not associated with an increased risk of MI.

We found that 18% of all-comers undergoing PCI at our institution had creatinine clearance <60 mL/min or at least moderate CKD by the National Kidney Foundation guidelines [14]. This number is not surprising considering that more than 8% of the adult population of the United States is estimated to have at least moderate CKD [17].

Several studies have shown that patients with CKD who undergo revascularization by PCI and stenting consistently have worse short- and long-term outcomes relative to patients without CKD [1], [2]. Many of these studies included patients who had PCI before the advent of DES. Studies that included patients with DES were small, single-centered, observational analyses. The follow-up period was usually 12 months or less with only 2 studies having follow-up longer than 12 months [18], [19]. There has been no randomized clinical trial investigating the efficacy of DES in patients with CKD.

We found that DES, compared with BMS, were associated with reduced risk of TVR. This finding echoes those of large studies of DES in the general population [6]. We found the use of DES to be a significant independent predictor of reduced all-cause mortality, compared to BMS. This reduction in mortality with DES was not accompanied by reductions in rates of MI or ST but was accompanied by lower rates of restenosis. The absolute benefits of DES compared to BMS may be greater in CKD patients given their higher restenosis risk, which can potentially contribute to the survival advantage. Patients with CKD, especially end-stage renal disease, have higher in-stent restenosis rates, irrespective of the type of stent [2]. Exaggerated neointimal growth in CKD patients has been attributed to higher rates of co-morbidities such as diabetes mellitus, greater atherosclerotic burden, vascular calcification, stent under expansion, chronic systemic inflammation, granulocyte activation and oxidative stress [2], [20], [21].

However, it is difficult to explain the lower mortality among DES patients solely by the reduced rates of restenosis. While patients receiving DES in our study were at higher risk with respect to rates of diabetes mellitus and hyperlipidemia, they were at lower risk with respect to rates of acute ST-segment elevation MI (STEMI), lesion characteristics, left ventricular function and success of PCI. Consequently, patients receiving DES in our study, by virtue of selection bias, had more stable coronary artery disease and lower baseline risk of thrombotic or restenotic events. The role of DES during PCI for STEMI has been controversial [22] and the lower use of DES in patients with acute MI reflects contemporary clinical practice.

Additionally, observational analyses are subject to confounding with respect to the nonrandomized choice of either DES or BMS. While multivariable adjustment and propensity matching mitigate the effect of measured confounders on the DES vs. BMS effect estimate, these approaches have limited ability to address the influence of unmeasured confounders. A survival benefit from DES has not been observed in randomized controlled trials. Thus, unmeasured confounders likely contribute to the reduction in mortality with DES in observational studies of DES vs. BMS use, including the current study. In a meta-analysis of 31 observational studies that included 169,595 all-comer patients, DES vs. BMS use was associated with an 18 to 22% reduction in mortality [6], but this effect was not noted in a meta-analysis of 21 randomized clinical trials that included 8,867 patients [6].

In the 2 studies of CKD patients with follow-up >12 months, use of DES compared to BMS reduced the risk of all-cause mortality at 17 months in the study by Zhang et al. [19], and did not reduce mortality at 4 years in the study by Appleby et al. [18]. While our findings are concordant with those of Zhang et al., they did not use any type of statistical designs (matching, covariate adjustment, or propensity-based adjustment) to adjust for differences between the DES and BMS patients. The discordance between our findings and those of Appleby et al. are likely due to differences in the clinical and angiographic characteristics of patients studied, duration of follow-up and potentially dissimilar unmeasured confounders. Of note, Appleby et al. found a significant survival benefit from DES compared to BMS in the first year (*p* = 0.002), with catch-up at 2 years (*p* = 0.057).

In the general population, DES are associated with an increased risk of late ST compared with BMS [23], [24], [25], [26]. However, we did not find a higher incidence of ST with DES relative to BMS at 3 years. This finding is notable since CKD has been described as a risk factor for ST after DES implantation [27], [28], [29]. Nonetheless, there have been no previous studies comparing the long-term incidence of ST with DES vs. BMS in patients with CKD. ST was not evaluated in the 2 studies of DES vs. BMS in CKD patients, with follow-up of >12 months [18], [19].

Two studies have compared ST at 12 months between DES and BMS in CKD patients [30], [31]. In the study by Halkin et al., there were no differences in the rates of ST between DES and BMS at 12 months in either patients with mild CKD (creatinine clearance 60 to 89 mL/min) or patients with at least moderate CKD (creatinine clearance <60 mL/min) [30]. Okada et al. only included patients on hemodialysis and found no significant difference in the rates of ST at 12 months between DES and BMS [31]. Our findings corroborate data from these 2 studies and suggest that there is no increase in the risk of ST at least up to 3 years after DES implantation in patients with creatinine clearance <60 mL/min. A possible explanation for this observation might be that the baseline endothelial dysfunction and inflammatory milieu in patients with CKD increases the risk of ST to similar degrees with DES and BMS.

### Limitations

As with any observational study, our study has the potential for unmeasured confounding. Creatinine clearance was assessed from a single measurement of pre-procedure creatinine. Therefore, the estimated creatinine clearance used in the study may not represent the true baseline renal function at the time of the index procedure and thus, misclassification bias is possible. Only first-generation DES were included in this study. To avoid confounding from multiple stent types, we excluded patients with prior PCI - a high-risk group of patients. We did not account for differences in the use of long-term medications such as antiplatelet therapy between the DES and BMS groups. The longer duration of dual antiplatelet therapy required with DES [32] may reduce long-term adverse event rates independently of stent selection [33]. We did not study outcomes stratified by every stage of CKD and for patients on dialysis. While it is possible that patients with stages 4 and 5 CKD may have worse outcomes relative to those of stage 3 CKD, and patients with stages 1 and 2 CKD may have worse outcomes relative to patients without CKD, our goal was to study the utility of DES in a broad group of patients with CKD that are at the greatest risk for atherosclerotic cardiovascular disease and need for revascularization, that is, patients with creatinine clearance <60 mL/min [1], [14]. Finally, we did not include data about the etiology of renal dysfunction in patients with creatinine clearance <60 mL/min.

### Summary

In a contemporary PCI registry, selective use of DES in patients with CKD was safe and effective in the long term, with lower risk of all-cause death, TVR and MACE and similar risk of MI and ST as compared with BMS. The mortality benefit may be a result of selection bias and residual confounding, or represent a true finding; a hypothesis that needs to be tested using randomized clinical trials. Given the increasing prevalence of CKD, the potential for improved ischemic outcomes and survival after PCI may have important implications for individual patients and for public health policy recommendations.
